# Variation in viscoelastic properties of bovine articular cartilage below, up to and above healthy gait-relevant loading frequencies

**DOI:** 10.1177/0954411915570372

**Published:** 2015-02

**Authors:** Hamid Sadeghi, Daniel M Espino, Duncan ET Shepherd

**Affiliations:** School of Mechanical Engineering, University of Birmingham, Birmingham, UK

**Keywords:** Articular cartilage, bovine, dynamic mechanical analysis, loss stiffness, mechanical properties, storage stiffness, viscoelasticity

## Abstract

The aim of this study was to determine the variation in viscoelastic properties of femoral head bovine articular cartilage, on-bone, over five orders of magnitude of loading frequency. These frequencies ranged from below, up to and above healthy gait-relevant frequencies, using<1, 1–5 and 10 Hz, respectively. Dynamic mechanical analysis was used to measure storage and loss stiffness. A maximum compressive force of 36 N was applied through a chamfered-end, 5.2-mm-diameter, indenter. This induced a maximum nominal stress of 1.7 MPa. The ratio of storage to loss stiffness increased from near parity (2.5) at low frequencies to 11.4 at 10 Hz. This was the result of a significant logarithmic increase (*p* < 0.05) in storage stiffness with frequency, from 367 N/mm (0.001 Hz) up to 1460 N/mm (10 Hz). In contrast, the loss stiffness remained approximately constant. In conclusion, viscoelastic properties of articular cartilage measured at frequencies below those of gait activities are poor predictors of its relevant dynamic mechanical behaviour.

## Introduction

In this study, dynamic mechanical analysis (DMA) has been used to determine the variation in viscoelastic properties of bovine articular cartilage, on-bone, over five orders of magnitude of frequency. Viscoelastic properties have been compared at loading frequencies associated with gait and at lower frequencies used experimentally. The findings highlight the limitations of extrapolating viscoelastic properties obtained at non-physiologically relevant frequencies for physiological function.

Articular cartilage is a load-bearing structure,^1,2^ which when undamaged contributes to smooth joint motion aided by a surface roughness of around 80–170 nm.^3^ However, osteoarthritis (OA) is associated with damaged cartilage and impaired or painful joint motion.^4^ Rapid heel-strike rise times during gait have been implicated in the early onset of OA in lower limb joints.^5,6^ However, heel-strike rise times not associated with OA are typically 100–150 ms^7^ and correspond to loading frequencies of 3–5 Hz.^8^

Viscoelastic properties of a material are characterised by storage and loss moduli.^9,10^ The storage modulus characterises the ability to store energy which is then available for elastic recoil. The loss modulus characterises the ability of the material to dissipate energy. Storage and loss moduli are calculated from the storage and loss stiffness, respectively, normalised using a shape factor which accounts for the dimensions of the sample.^8,11^

DMA has been used to determine the viscoelastic properties of articular cartilage, on-bone, ranging from a standard walking pace (1 Hz), to healthy gait heel-strike relevant frequencies (3–5 Hz) and up to frequencies associated with traumatic heel-strike rates (90 Hz).^8,12–14^ However, most studies characterise cartilage within the range of 0.1–10 Hz^15–17^ with some studies doing so only at individual frequencies, for example, 0.1,^18,19^ 1^20,21^ and 3 Hz.^22^ At the nano-scale, large variability in viscoelastic properties of articular cartilage has already been observed over several orders of magnitude of frequency.^23,24^ Tanaka et al.^25^ reported on the viscoelastic properties of mandibular cartilage, on-bone, over frequencies ranging between 0.01 and 10 Hz. However, the variation in viscoelastic properties over a similar frequency range for lower limb joint articular cartilage, on-bone, has not been determined. Thus, limitations associated with determining viscoelastic properties obtained at below gait-relevant frequencies or extrapolating cartilage viscoelastic behaviour from a single frequency remain unclear.

This study aimed to determine articular cartilage viscoelastic properties below, up to and above frequencies associated with healthy gait cycles. The range of frequencies applied covers five orders of magnitude. Bovine cartilage was used as it is an accepted model for human cartilage and of similar thickness.^26,27^ DMA was used to measure storage and loss stiffness, with cartilage thickness measured so that respective moduli can be easily derived.

## Methods

### Specimens

Three bovine femoral heads, approximately between 18 and 30 months old, were obtained from a supplier (Johnston’s Butcher, Kings Heath, Birmingham, UK), consistent with previous studies.^12,13^ Further information regarding the animal from which samples were taken was not available. Upon arrival in the laboratory, samples were wrapped in tissue paper, saturated in Ringer’s solution, sealed in plastic bags and stored in a freezer at −40 °C. Prior to testing, samples were thawed, and a test specimen was obtained. Such freeze–thaw treatment does not alter the dynamic mechanical properties of cartilage.^14^

Each femoral head was dissected in half, with up to three suitable test regions being identified on each specimen for testing. Samples included subchondral bone (see section ‘DMA frequency sweep’, for cartilage thickness measurement). The underlying bone prevents cartilage swelling.^28^ Pre-existing surface lesions were identified with India ink (Loxley Art Materials, Sheffield, UK).^29^ Only intact surfaces were tested, as surface cracks alter the mechanical properties of cartilage.^30^

### DMA frequency sweep

**Figure 1.**
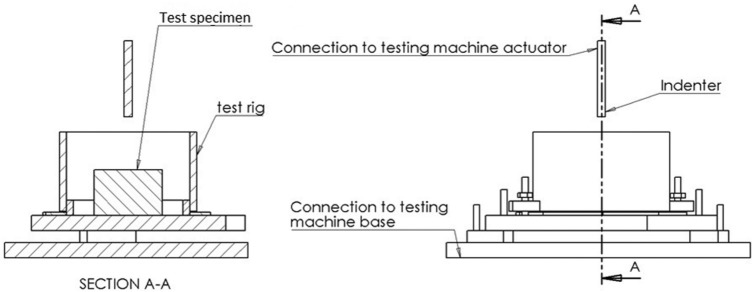
Diagram of the experimental set-up used during the testing procedure. The set-up includes a device which contains a segment of the femoral head, fixed in place using acrylic cement.

**Figure 2.**
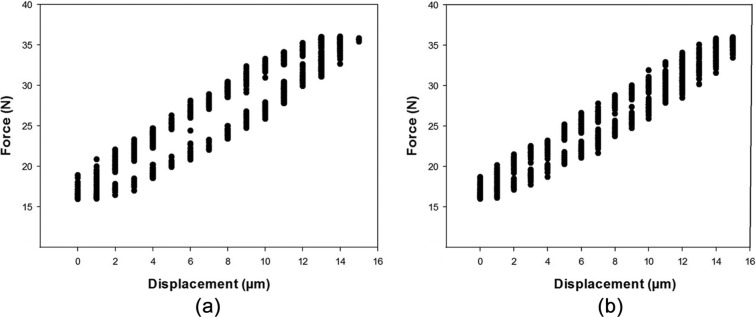
Representative experimental data of the measured force and displacement, from multiple cycles, for a given sample at (a) 0.01 and (b) 1 Hz.

The experimental protocol has been defined and used to test lower limb cartilage previously^8,12,13^ (Figure 1). Briefly, samples were secured in a custom-made rig with acrylic polymer cement (WHW Plastics, Hull, UK) bathed in Ringer’s solution at room temperature. The apparatus was secured to the base of the testing machine and enabled small adjustments. Hence, the surface of the articular cartilage being tested was oriented perpendicular to the indenter’s direction of compression. WinTest DMA software (Bose Corporation, Eden Prairie, MN) was used to control a material testing machine (Bose ElectroForce 3200). A nominal compressive stress was induced by applying a sinusoidally varying compressive force between 16 and 36 N. Loads were applied using a cylindrical indenter (diameter of 5.2 mm). The indenter has a chamfered end to prevent cartilage damage at the contact area edge. The loading range used induced deformation, as shown in Figure 2, and resulted in dynamic strains of around 1%, comparable to a previous study.^8^ Peak loading induced maximum stresses of up to 1.7 MPa. These peak stresses have been estimated to be physiological for lower limb human articular cartilage.^31^

A total of 18 DMA frequency sweep tests were performed at frequencies of 0.001, 0.01, 0.1, 1 and 10 Hz, corresponding to five orders of magnitude of frequencies. Two preload conditions were applied before the frequency sweep, at 25 and 50 Hz (1500 and 3000 cycles, respectively, with a 60-s rest period), following recommendation from previous studies.^8,13^ Such precycling is consistent with cartilage requiring over 1200^32^ or 2000^33^ loading cycles to reach a steady state.

Cartilage thickness was measured after the final test using a previously described technique.^7,8^ Briefly, a sharp needle is pushed through the cartilage layer and up to the bone using a testing machine. The thickness of all samples tested is included in Table 1.

**Table 1.** Summary of the results for storage stiffness described by equation (1) and mean loss stiffness.

Storage stiffness coefficients						Loss stiffness (N/mm)
Point	Thickness (mm)	*A* (N/mm)	*B* (N/mm)	*R* ^2^	*p*	Mean ± SD
1	2.1	120	1249	0.97	<0.05	217 ± 30
2	2.5	117	1459	0.94	<0.05	179 ± 37
3	2.0	125	1572	0.94	<0.05	193 ± 42
4	2.1	125	1173	0.97	<0.05	189 ± 48
5	2.7	114	1137	0.96	<0.05	180 ± 41
6	2.2	75	970	0.92	<0.05	147 ± 28
7	1.7	164	1907	0.87	<0.05	218 ± 86
8	2.1	164	1937	0.84	<0.05	213 ± 97
9	1.9	118	1503	0.85	<0.05	199 ± 63
10	2.2	80	846	0.90	<0.05	114 ± 41
11	2.4	83	882	0.91	<0.05	119 ± 43
12	2.0	71	733	0.91	<0.05	101 ± 38
13	2.1	142	1612	0.82	<0.05	175 ± 57
14	2.4	141	1729	0.85	<0.05	176 ± 63
15	2.2	95	1207	0.91	<0.05	158 ± 40
16	1.9	89	998	0.94	<0.05	127 ± 41
17	1.6	111	1262	0.94	<0.05	175 ± 51
18	2.1	104	1216	0.94	<0.05	159 ± 49
Mean ± SD	2.1 ± 0.3	113 ± 28	1300 ± 357	–	–	169 ± 36

SD: standard deviation.

### Viscoelastic data analysis

The applied force and resulting displacement were measured at each individual frequency and used to calculate the dynamic stiffness, k* (i.e. ratio of force to displacement) and the phase angle, *δ*, between the force and displacement.^34^ The storage, *k*′, and loss, *k*″, stiffness were obtained from the dynamic stiffness and phase angle, as *k*′ = k*cos *δ* and *k*″ = k* sin *δ*, which is described in further detail elsewhere.^11,34^ As the indenter diameter is constant, the only geometric variable was the sample thickness, which does not vary for an individual point tested over a range of frequencies. Storage stiffness was plotted against frequency, and a curve was fitted to the data in the form

(1) $$k\prime =A\log_{e}\left(f\right)+B$$

where *A* defines the gradient of *k*′ plotted against the natural logarithm of *f*, the loading frequency (Hz), and *B* is the intercept. The two constants, *A* and *B*, are used to characterise the frequency-dependent storage stiffness of samples.^13^ A similar curve fit has been used for the storage modulus previously.^8^ Regression analysis was used to determine whether the relationship was significant for all samples tested. Note that modulus (storage, loss, complex) can be calculated from the equivalent stiffness and a shape factor, *S*, dependent on indenter diameter and sample thickness.^8^

The storage and loss stiffness of a viscoelastic structure can be represented using an Argand diagram. The loss stiffness lies on an imaginary axis and the storage stiffness on the real axis. The complex stiffness, k* (equation 2), and phase angle, *δ* (equation 3), are related to the storage and loss stiffness

(2) $$\left|k^{*}\right|=\sqrt{k\prime^{2}+{k\prime \prime }^{2}}$$

(3) $$\delta =\tan^{-1}\left(\frac{k\prime \prime }{k\prime }\right)$$

## Results

### Frequency dependency

**Figure 3.**
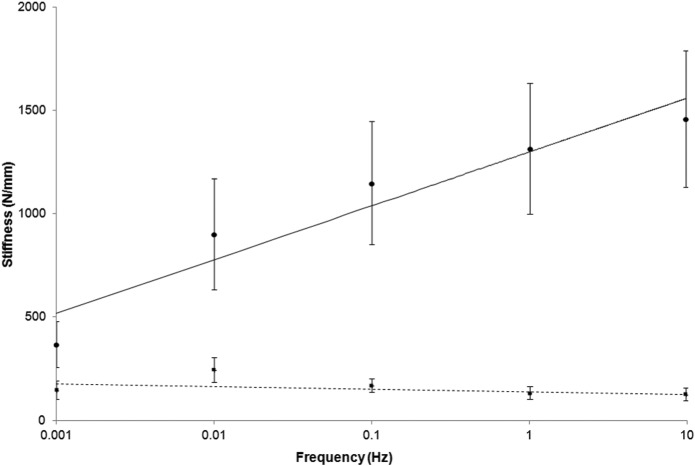
Logarithmic frequency dependency of the mean storage (circles, solid line) and loss (squares, dashed line) stiffness (including standard deviation as error bars) at loading frequencies between 0.001 and 10 Hz.

**Figure 4.**
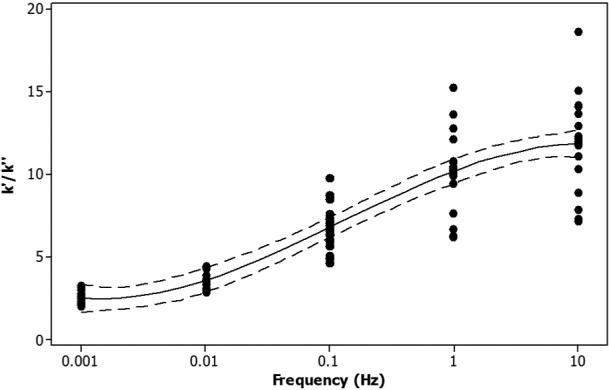
Ratio of storage (*k*′) to loss (*k*″) stiffness against the logarithm of the frequency. All data points are included (black circles), along with the mean trendline (solid line) and 95% confidence intervals (dashed line). Note that 95% confidence intervals refer to the reliability of the procedure used and not to the likelihood that an individual data point will appear within that range or that 95% of data points are found within that range.^35^

The frequency dependency of the storage and loss stiffness for all samples tested, over five orders of magnitude of frequency, is shown in Figure 3. The storage stiffness was always greater than the loss stiffness. At higher frequencies, the storage stiffness was approximately an order of magnitude greater than the loss stiffness (Figure 3). However, at lower frequencies, the storage-to-loss stiffness ratio tended towards parity. For example, at 0.001 Hz, the storage-to-loss stiffness ratio was 2.5, but it was 11.4 at 10 Hz. This ratio increased significantly with frequency (Figure 4; *p* < 0.05, *R*^2^ = 0.81) as a consequence of the variation in storage stiffness with frequency.

The storage stiffness increased with frequency, from 367 ± 112 N/mm at 0.001 Hz up to 1460 ± 331 N/mm at 10 Hz (Table 2). Storage stiffness increased linearly with the logarithm of the frequency (Figure 3). This relationship was described using two constants, *A* and *B* (see section ‘Viscoelastic data analysis’). The mean value for constant *A* was 113 ± 28 N/mm and for the intercept *B* 1300 ± 357 N/mm (Table 1). From regression analysis, this trend was found to be significant for all samples tested (*p* < 0.05; 0.97 ≥ *R*^2^ ≥ 0.82; Table 1).

**Table 2.** Mean storage, *k*′, and loss, *k*″, stiffness at different frequencies, *f*.

*f* (Hz)	*k*′ (N/mm); mean ± SD	*k*″ (N/mm); mean ± SD	*k*′*/k*″
0.001	367 ± 112	148 ± 53^A^	2.5
0.01	902 ± 268	247 ± 65^B^	3.7
0.1	1149 ± 298	170 ± 37^A^	6.8
1	1315 ± 317	133 ± 35^A^	9.9
10	1460 ± 331	128 ± 37^A^	11.4

SD: standard deviation.

The letters A and B are used to indicate significant differences between loss stiffness at different frequencies; where a frequency does not share a letter they are significantly different (*p* < 0.05).

The loss of stiffness of articular cartilage, on-bone, was mostly frequency independent (Figure 3) with a mean of 169 ± 36 N/mm (Table 1). However, at 0.01 Hz, the loss stiffness increased significantly to 247 ± 65 N/mm (*p* < 0.05; Table 2) compared to a mean loss stiffness of 145 ± 43 N/mm for all other frequencies.

### Phase angle and complex stiffness

**Figure 5.**
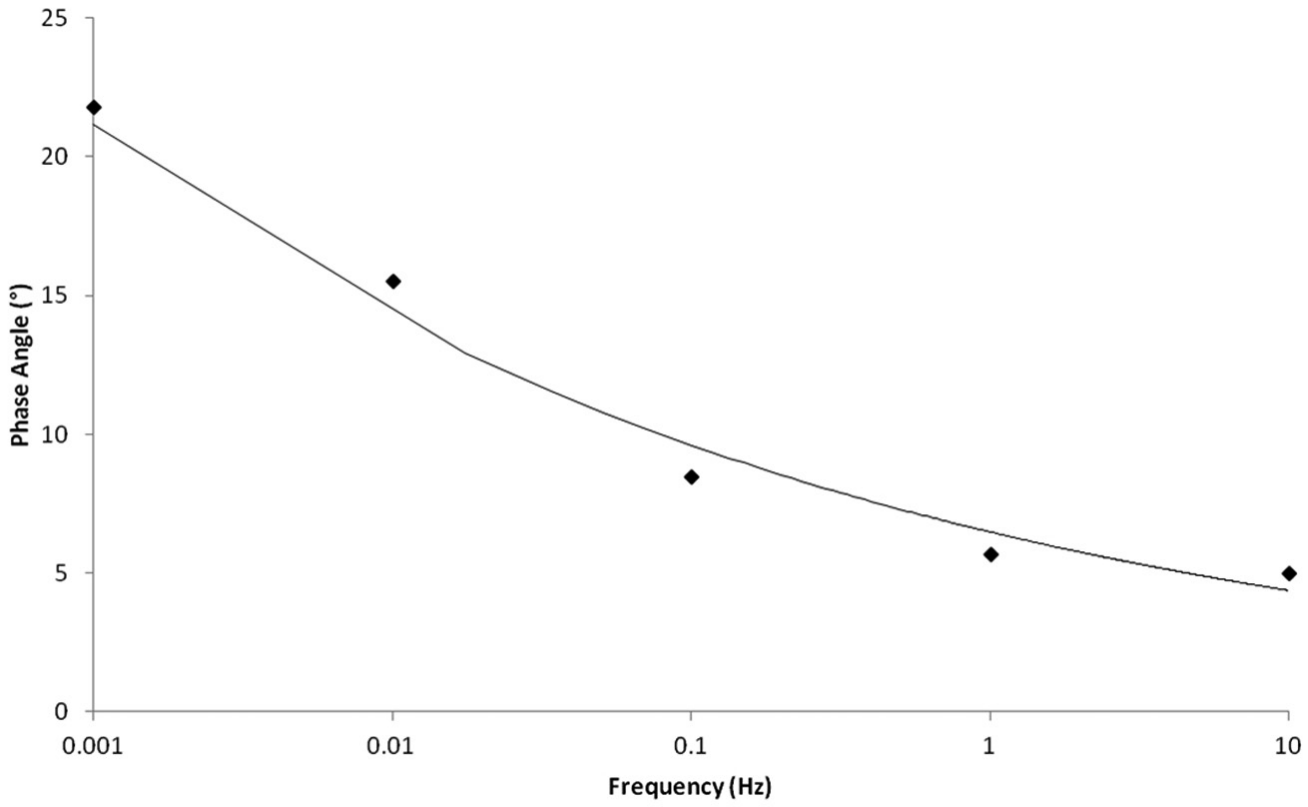
Change in phase angle with loading frequency. For consistency with other figures, a logarithmic plot is presented. However, the empirical relationship between the phase angle and frequency is best described through a power function (equation (4)), as determined using regression analysis.

Large changes in the phase angle occurred at frequencies below 1 Hz (Figure 5). Regression analysis of the data obtained showed that there was an empirical relationship between the mean phase angle, *δ*, and the frequency, *f*. It was found that *δ* decreased as a power function of *f* (equation (4), *R*^2^ = 0.96). It decreased from 22° at 0.001 Hz and tended towards an asymptote of 5° as the loading frequency increased towards 10 Hz

(4) $$\delta =6.4851f^{-0.1711}$$

**Figure 6.**
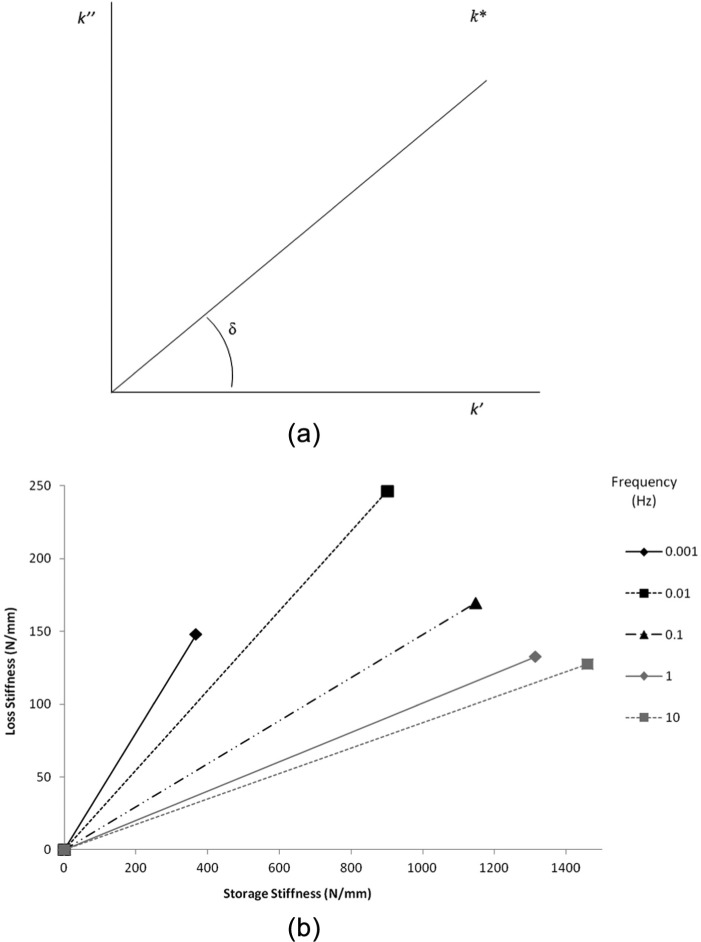
Variation in storage and loss stiffness of articular cartilage with increasing loading frequency. (a) Argand diagram including phase angle (*δ*), complex (k*), storage (*k*′) and loss (*k*″) stiffness. (b) Variation in *k*′ and *k*″ with frequency.

At lower frequencies, there were large changes in the complex stiffness. The complex stiffness increased by 58% from 0.001 Hz (396 N/mm) to 0.01 Hz (935 N/mm), thereafter only increasing by 36% over a four orders of magnitude increase in loading frequency (i.e. at 10 Hz, k* = 1466 N/mm). Limitations of extrapolating from low-frequency testing to predicting physical behaviour which occurs at higher frequencies can be interpreted visually from Figure 6.

## Discussion

### Key findings

DMA has been used to characterise the storage and loss stiffness of bovine articular cartilage, on-bone, over five orders of magnitude of frequency. The findings from this study demonstrate that it is necessary to measure mechanical properties of articular cartilage over physiological frequencies. Measuring properties at low loading frequencies predicts viscoelastic properties not representative of articular cartilage mechanical behaviour during gait activities. This is because of the sensitivity of the storage stiffness to the loading frequency. However, the loss stiffness was frequency independent.

Some studies report phase angles and complex moduli (or stiffness) instead of storage and loss moduli or stiffness.^14^ Calculation of a phase angle and complex stiffness demonstrate that below 1 Hz, there are large changes in the phase angle with large changes to the complex stiffness occurring below 0.01 Hz. Therefore, studies investigating articular cartilage at frequencies below 1 Hz, but above 0.01 Hz, would report a gait-relevant complex stiffness (and/or modulus) but not phase angle.

### Storage stiffness

In this study, the storage stiffness was found to increase with frequency. Therefore, determining viscoelastic properties at a single loading frequency ignores this dependency. Moreover, at frequencies below 1 Hz, the storage stiffness was much lower than at gait-relevant loading frequencies and so not appropriate for the study of articular cartilage under physiological conditions.

The frequency-dependent increase in storage stiffness led to an increase in the storage to loss stiffness ratio from 2.5 to 11.4. This is consistent with the ratio being near parity following impact tests.^36^ The frequency-dependent ratio increase implies that excess energy is stored in cartilage with increased frequency.^8^ Such energy could potentially be dissipated through failure, such as the formation of cracks, and is consistent with impulsive heel-strike rise times being associated with the early onset of OA.^6^ Low frequencies may also lead to errors if being used to predict cartilage failure.^19^ In this study, no visible signs of damage were observed following testing, not unexpected as the maximum induced stress was 1.7 MPa. Articular cartilage failure stress is in the region of 8–10 MPa.^1,2^

### Loss stiffness

A frequency-independent loss stiffness may not appear consistent with increased hysteresis with loading velocity for off-bone cartilage^36^ or the frequency-dependent increase in loss modulus for some polymers.^34^ However, restriction caused by bone^37^ has been predicted to prevent an increase in loss modulus with frequency,^36^ consistent with previous findings^8,13^ and our current experimental findings.

The lowest frequencies used in this study were anticipated to enable sufficient time for any fluid dissipative effects to occur. For example, 0.001 Hz has a period of 16 min 40 s, which is of comparable duration to the 15 min of loading required for peak pore pressure to develop.^1^ The phenomenon of peak pore pressure rise in cartilage has previously been used to explain its failure.^1,2^ Under such a model, a greater loss stiffness would be expected at lower frequencies. However, this is not supported by our current results, as the loss stiffness is frequency independent, which is consistent with previous findings at higher frequencies.^8^ Thus, the interaction between collagen and gel-matrix^38^ including stress transfer mechanisms^39–42^ is expected to determine the conservation and dissipation of energy in cartilage. A fibril-reinforced model used to model cartilage at the nano-scale has already led to good agreement with the results from dynamic loading.^23^

### Relation to static loading

Load rise times can be estimated from loading frequencies, with time equal to the inverse of twice the frequency.^8^ Therefore, loading frequencies of 0.01 and 1 Hz are equivalent to loading times of 50 and 0.5 s, respectively. Note, these are the frequencies between which a complex modulus (or stiffness), but not phase angle, would lead to gait-relevant values. Hence, mechanical properties representative of physical behaviour during gait-relevant activities requires loading within 0.5 s, consistent with previous studies.^7^ The implication is that ultra-structural assessment of articular cartilage following extended loading (e.g. tissue fixation) may not represent interactions between collagen and gel under gait-relevant loading. This is because under extended loading, the physical mechanism by which energy is stored in the tissue to enable subsequent recoil is not equivalent to that which occurs at higher loading rates.

Viscoelastic materials have a characteristic relaxation time, ***τ***, which can be used to describe their mechanical behaviour during single-load or single-strain experiments. Examples of these are creep and stress relaxation:^43^ during creep testing, the extension measured for cartilage would increase over time and reach a plateau, whereas during stress relaxation, the stress would asymptote through exponential decay. ***τ*** is proportional to the ratio of loss to storage stiffness.^34^ Our results show that this ratio can change by an order of magnitude, which would result in large differences in predictions of creep and stress relaxation. For example, a low value of ***τ*** would lead to a rapid transition towards an asymptotic stress or strain, whereas a high value of ***τ*** would heavily dampen the transition to asymptotic stress or strain. Therefore, the longer relaxation time, which would be derived from low frequencies of loading, is only likely to approximate the viscoelastic behaviour of cartilage loaded over a long time period. These predictions would be relevant to studies which last for long enough to enable the development of peak pore pressure (i.e. ≥15 min of loading)^44^ but would not be relevant to the mechanical behaviour of articular cartilage during gait-relevant loading.^8^ Thus, when inferring the mechanical behaviour of cartilage, appropriate loading protocols must be used, in particular if aiming to make predictions relevant to gait activities.

A possible limitation of this study is the use of a linearly viscoelastic definition for articular cartilage. However, if the storage and loss moduli (or stiffness) of a material are constant, or vary only with time, then the material is linearly viscoelastic.^34^ This is consistent with the storage stiffness of cartilage being frequency dependent (equation (1)).^8,12,13^ Furthermore, for a sufficiently small displacement, any viscoelastic material will be effectively linearly viscoelastic. In this study, the dynamic strain was of the order of 1%, while inducing a physiologically relevant stress. Therefore, under the experimental conditions used for this study, viscoelastic characterisation of cartilage is suitable and consistent with previous studies.^8,9,12–14^

## Conclusion

Viscoelastic properties of articular cartilage measured at frequencies below those of gait activities are poor predictors of its relevant dynamic mechanical behaviour.

## References

[1] Fick JM Espino DM Articular cartilage surface rupture during compression: investigating the effects of tissue hydration in relation to matrix health. J Mech Behav Biomed Mater 2011; 4: 1311–1317. 21783140 10.1016/j.jmbbm.2011.04.018

[2] Fick JM Espino DM Articular cartilage surface failure: an investigation of the rupture rate and morphology in relation to tissue health and hydration. Proc IMechE, Part H: J Engineering in Medicine 2012; 226: 389–396. 10.1177/0954411912439824 22720392

[3] Ghosh S Bowen J Jiang K Investigation of techniques for the measurement of articular cartilage surface roughness. Micron 2013; 44: 179–184. 22771276 10.1016/j.micron.2012.06.007

[4] Felson DT Lawrence RC Dieppe PA Osteoarthritis: new insights. Part 1: the disease and its risk factors. Ann Intern Med 2000; 133: 635–646. 11033593 10.7326/0003-4819-133-8-200010170-00016

[5] Radin EL Whittle MW Yang KH The heelstrike transient, its relationship with the angular velocity of the shank, and effects of quadriceps paralysis. In Lantz SA King AI (eds) Advances in bioengineering. New York: American Society of Mechanical Engineering, 1986, pp.121–123.

[6] Radin EL Yang KH Riegger C Relationship between lower limb dynamics and knee-joint pain. J Orthop Res 1991; 9: 398–405. 2010844 10.1002/jor.1100090312

[7] Shepherd DET Seedhom BB Technique for measuring the compressive modulus of articular cartilage under physiological loading rates with preliminary results. Proc IMechE, Part H: J Engineering in Medicine 1997; 211: 155–165. 10.1243/0954411971534278 9184456

[8] Fulcher GR Hukins DWL Shepherd DET Viscoelastic properties of bovine articular cartilage attached to subchondral bone at high frequencies. BMC Musculoskelet Disord 2009; 10: 61. 19497105 10.1186/1471-2474-10-61 PMC2698871

[9] Aspden RM Aliasing effects in Fourier transforms of monotonically decaying functions and the calculation of viscoelastic moduli by combining transforms over different time periods. J Phys D Appl Phys 1991; 24: 803–808.

[10] Hukins DWL Leahy JC Mathias KJ Biomaterials: defining the mechanical properties of natural tissues and selection of replacement materials. J Mater Chem 1999; 9: 629–636.

[11] Wilcox AG Buchan KG Espino DM Frequency and diameter dependent viscoelastic properties of mitral valve chordae tendineae. J Mech Behav Biomed Mater 2014; 30: 186–195. 24316874 10.1016/j.jmbbm.2013.11.013

[12] Espino DM Shepherd DET Hukins DWL Viscoelastic properties of bovine knee joint articular cartilage: dependency on thickness and loading frequency. BMC Musculoskelet Disord 2014; 15: 205. 24929249 10.1186/1471-2474-15-205 PMC4068975

[13] Pearson B Espino DM The effect of hydration on the frequency-dependent viscoelastic properties of articular cartilage. Proc IMechE, Part H: J Engineering in Medicine 2013; 227: 1246–1252. 10.1177/0954411913501294 23982065

[14] Szarko M Muldrew K Bertram JEA Freeze–thaw treatment effects on the dynamic mechanical properties of articular cartilage. BMC Musculoskelet Disord 2010; 11: 231. 20932309 10.1186/1471-2474-11-231 PMC2958988

[15] Fortis AP Kostopoulos V Panagiotopoulos E Viscoelastic properties of cartilage-subchondral bone complex in osteoarthritis. J Med Eng Technol 2004; 28: 223–226. 15371002 10.1080/03091900410001676003

[16] Schwartz CJ Bahadur S Investigation of articular cartilage and counterface compliance in multi-directional sliding as in orthopedic implants. Wear 2007; 262: 1315–1320.

[17] Ronken S Arnold MP Ardura-Garcia H A comparison of healthy human and swine articular cartilage dynamic indentation mechanics. Biomech Model Mechanobiol 2012; 11: 631–639. 21769620 10.1007/s10237-011-0338-7

[18] Ronken S Arnold MP Hoechel S Mapping of dynamic stiffness properties of cartilage on the human patella. J Biomech 2012; 45(Suppl. 1): S157.

[19] Weightman BO Freeman MA Swanson SA Fatigue of articular cartilage. Nature 1973; 244: 303–304. 4270399 10.1038/244303a0

[20] Kurkijärvi JE Nissi MJ Kiviranta I Delayed gadolinium-enhanced MRI of cartilage (dGEMRIC) and T2 characteristics of human knee articular cartilage: topographical variation and relationships to mechanical properties. Magn Reson Med 2004; 52: 41–46. 15236365 10.1002/mrm.20104

[21] Gannon AR Nagel T Kelly DJ The role of the superficial region in determining the dynamic properties of articular cartilage. Osteoarthritis Cartilage 2012; 20: 1417–1425. 22890186 10.1016/j.joca.2012.08.005

[22] Stolz M Raiteri R Daniels AU Dynamic elastic modulus of porcine articular cartilage determined at two different levels of tissue organization by indentation-type atomic force microscopy. Biophys J 2004; 86: 3269–3283. 15111440 10.1016/S0006-3495(04)74375-1 PMC1304192

[23] Nia HT Han L Li Y Poroelasticity of cartilage at the nanoscale. Biophys J 2011; 101: 2304–2313. 22067171 10.1016/j.bpj.2011.09.011 PMC3207157

[24] Han L Frank EH Greene JJ Time-dependent nanomechanics of cartilage. Biophys J 2011; 100: 1846–1854. 21463599 10.1016/j.bpj.2011.02.031 PMC3072655

[25] Tanaka E Yamano E Dalla-Bona DA Dynamic compressive properties of the mandibular condylar cartilage. J Dent Res 2006; 85: 571–575. 16723658 10.1177/154405910608500618

[26] Kääb MJ Ap Gwynn I Nötzli HP Collagen fibre arrangement in the tibial plateau articular cartilage of man and other mammalian species. J Anat 1998; 193: 23–34. 9758134 10.1046/j.1469-7580.1998.19310023.x PMC1467820

[27] Taylor SD Tsiridis E Ingham E Comparison of human and animal femoral head chondral properties and geometries. Proc IMechE, Part H: J Engineering in Medicine 2012; 226: 55–62. 10.1177/0954411911428717 22888585

[28] Summers GC Merrill A Sharif M Swelling of articular cartilage depends on the integrity of adjacent cartilage and bone. Biorheology 2008; 45: 365–374. 18836237

[29] Meachim G Light microscopy of Indian ink preparations of fibrillated cartilage. Ann Rheum Dis 1972; 31: 457–464. 4117785 10.1136/ard.31.6.457 PMC1005976

[30] Burgin LV Aspden RM Impact testing to determine the mechanical properties of articular cartilage in isolation and on bone. J Mater Sci Mater Med 2008; 19: 703–711. 17619965 10.1007/s10856-007-3187-2

[31] Yao JQ Seedhom BB Mechanical conditioning of articular cartilage to prevalent stress. Br J Rheumatol 1993; 32: 956–965. 8220934 10.1093/rheumatology/32.11.956

[32] Verteramo A Seedhom BB Effect of a single impact loading on the structure and mechanical properties of articular cartilage. J Biomech 2007; 40: 3580–3589. 17662988 10.1016/j.jbiomech.2007.06.002

[33] McCormack T Mansour JM Reduction in tensile strength of cartilage precedes surface damage under repeated compressive loading in vitro. J Biomech 1998; 31: 55–61. 9596538 10.1016/s0021-9290(97)00103-6

[34] Haddad YM Viscoelasticity of engineering materials. 1st ed. London: Chapman & Hall, 1995.

[35] Bland M An introduction to medical statistics. 3rd ed. Oxford: Oxford University Press, 2000.

[36] Edelsten L Jeffrey JE Burgin LV Viscoelastic deformation of articular cartilage during impact loading. Soft Matter 2010; 6: 5206–5212.

[37] Aspden RM Constraining the lateral dimensions of uniaxially loaded materials increases the calculated strength and stiffness: application to muscle and bone. J Mater Sci Mater Med 1990; 1: 100–104.

[38] Lewis RJ MacFarland AK Anandavijayan S Material properties and biosynthetic activity of articular cartilage from the bovine carpo-metacarpal joint. Osteoarthritis Cartilage 1998; 6: 383–392. 10343771 10.1053/joca.1998.0142

[39] Aspden RM Fibre reinforcing by collagen in cartilage and soft connective tissues. Proc Biol Sci 1994; 258: 195–200. 7838856 10.1098/rspb.1994.0162

[40] Goh KL Mathias KJ Aspden RM Finite element analysis of the effect of fibre shape on stresses in an elastic fibre surrounded by a plastic matrix. J Mater Sci 2000; 35: 2493–2497.

[41] Goh KL Meakin JR Aspden RM Influence of fibril taper on the function of collagen to reinforce extra-cellular matrix. Proc Biol Sci 2005; 272: 1979–1983. 16191606 10.1098/rspb.2005.3173 PMC1559877

[42] Goh KL Meakin JR Hukins DWL Influence of fibre taper on the interfacial shear stress in fibre-reinforced composite materials during elastic stress transfer. Compos Interface 2010; 17: 74–80.

[43] Holmes AD Hukins DWL Analysis of load-relaxation in compressed segments of lumbar spine. Med Eng Phys 1996; 18: 99–104. 8673325 10.1016/1350-4533(95)00047-x

[44] Fick JM How the structural integrity of the matrix can influence the microstructural response of articular cartilage to compression. Connect Tissue Res 2013; 54: 83–93. 23126382 10.3109/03008207.2012.746321

